# Effectiveness of Checklist-Based Box System Interventions (CBBSI) versus routine care on improving utilization of maternal health services in Northwest Ethiopia: study protocol for a cluster randomized controlled trial

**DOI:** 10.1186/s13063-019-4002-3

**Published:** 2020-02-07

**Authors:** Netsanet Belete Andargie, Mulusew Gerbaba Jebena, Gurmesa Tura Debelew

**Affiliations:** 1 Ministry of Health, Addis Ababa, Ethiopia; 2 Department of Population and Family Health, Jimma University, Jimma, Ethiopia

**Keywords:** Box system, Maternal health, Antenatal care, Skilled delivery, Postnatal care, Cluster randomized controlled trial, Ethiopia

## Abstract

**Background:**

Maternal mortality is still high in Ethiopia. Antenatal care, the use of skilled delivery and postnatal care are key maternal health care services that can significantly reduce maternal mortality. However, in low- and middle-income countries, including Ethiopia, utilization of these key services is limited, and preventive, promotive and curative services are not provided as per the recommendations. The aim of this study is to examine the effectiveness of checklist-based box system interventions on improving maternal health service utilization.

**Methods:**

A community-level, cluster-randomized controlled trial will be conducted to compare the effectiveness of checklist-based box system interventions over the routine standard of care as a control arm. The intervention will use a health-extension program provided by health extension workers and midwives using a special type of health education scheduling box placed at health posts and a service utilization monitoring box placed at health centers. For this, 1200 pregnant mothers at below 16 weeks of gestation will be recruited from 30 clusters. Suspected pregnant mothers will be identified through a community survey and linked to the nearby health center. With effective communication between health centers and health posts, dropout-tracing mechanisms are implemented to help mothers resume service utilization. Data will be collected using an open data kit and analyzed using STATA version 13.0. Data will be analyzed by the intention-to-treat analysis. Risk ratios will be computed at the cluster level and the summary will be compared using *t* tests. Outcomes between intervention and control groups will be compared with random effects logistic regression models. Achieving four antenatal care visits, health facility delivery, and postnatal care visits at 6 weeks after delivery were treated as primary outcomes for this study.

**Discussion:**

We expect that the study will generate evidence on the effectiveness of checklist-based box system interventions on improving utilization of maternal health care service that will produce inputs for related policies in Ethiopia.

**Trial registration:**

ClinicalTrials.gov, NCT03891030. Retrospectively registered on 26 March 2019.

## Background

Under-utilization of the key maternal health services of antenatal care (ANC), use of skilled delivery attendants and postnatal care (PNC) contribute to a high rate of maternal mortality in developing countries. Global health goals still offer a renewed opportunity to see improvements in maternal health [1–3]. It has been argued that attending the specified services on a continuum of care perspective provides a platform for important health care functions, including health promotion, screening and diagnosis, and disease prevention [4–6].

In Ethiopia, one explanation for poor health outcomes among women is the lack of use of modern health care services by a great proportion of women [7]. Even though the overall utilization is low, women are more likely to attend for ANC than to deliver in health facilities [8–11]. This is evidenced by the result of the recently conducted Ethiopian Mini-Demographic and Health survey in 2019; 43% of mothers had four or more ANC visits while less than half of mothers (48%) gave birth in health facilities, and PNC within 2 days of delivery was sought by 34% of mothers [10]. In addition, early entry to ANC would improve the number of consecutive visits that a woman made [12]. However, the analysis of the 2011 Ethiopian Demographic and Health Survey showed that only 26.2% of those attending ANC started their ANC visit during the recommended time (first trimester), with the majority (56.4%) starting during the second trimester and 16.7% starting during the third trimester [2].

In recognition of these problems, the government of Ethiopia identified maternal health services as a key component of the country’s flagship health-extension program [13]. However,, limited intervention studies were conducted on improving maternal health services, and most were m-health based, which require a mother or her relative to own a mobile phone [14]. Some of these studies reported effectiveness in improving maternal health service utilization [15].

A checklist-based box system intervention (CBBSI) was designed with the aim of increasing utilization of maternal health services that could be rolled out both in urban and rural settings. The boxes were designed to fit with the currently implemented focused ANC approach for ANC and the World Health Organization (WHO) recommendation on PNC visits. The basic intention of the trial is to create demand for service utilization and provide dropout tracing through monitoring health service utilization.

## Methods/design

### Design

**Fig. 1:**
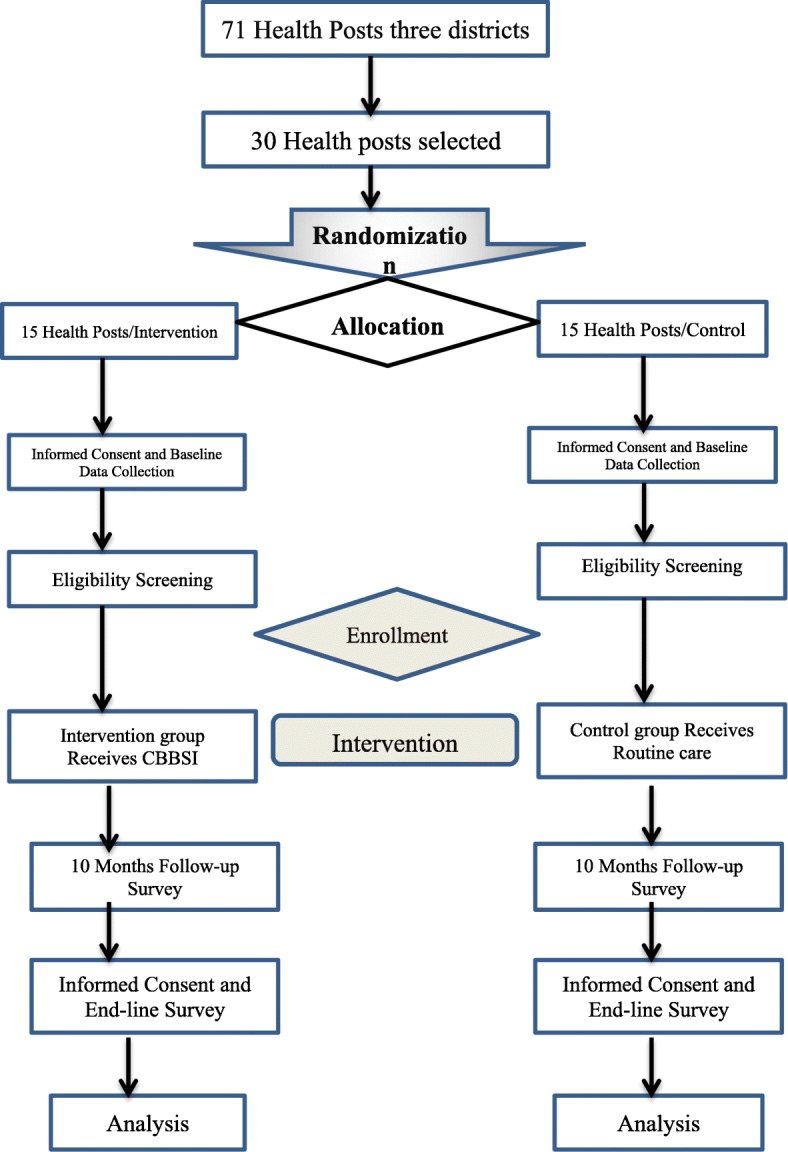
Participant flow through the trial. CBBSI checklist-based box system intervention


The study is a double-blind, parallel-group, two-arm cluster randomized controlled trial to assess the effectiveness of CBBSI on improving utilization of maternal health care services in East Gojjam Zone, northwest Ethiopia. The participant flow details are shown in Fig. 1.

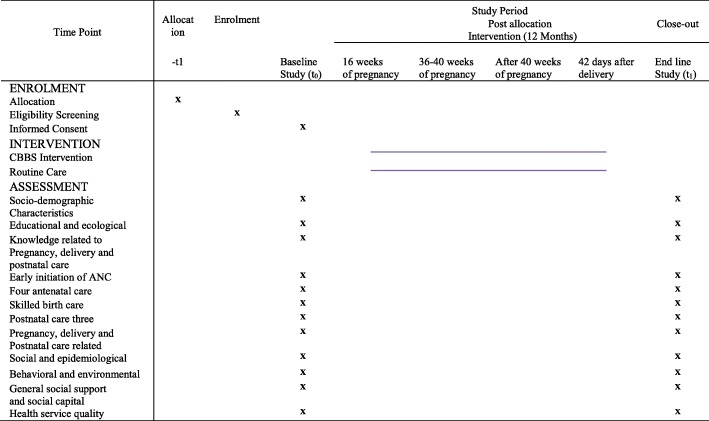

**Table 1:**
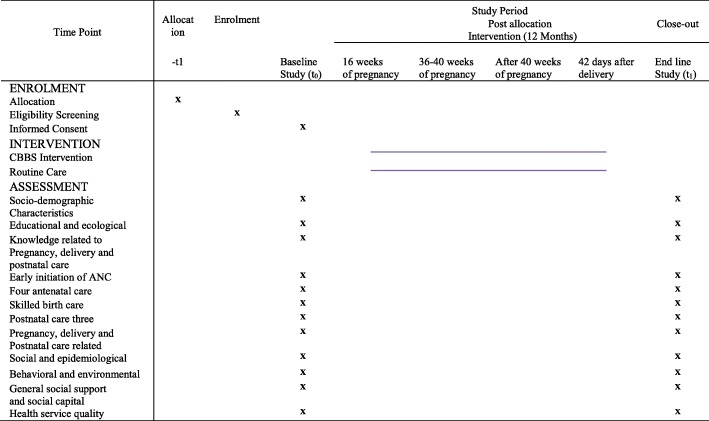
Representation of a checklist-based box system intervention using Standard Protocol Items: Recommendations for Interventional Trials (SPIRIT)

*ANC* antenatal care

### Setting

The study will be conducted in East Gojjam zone, one of the administrative zones of the Amhara region in northwestern Ethiopia. According to the 2016 Ethiopian Demographic and Health Survey, the region’s maternal health service utilization for the first and fourth ANC was found to be 67.1% and 31.5%, respectively, health facility delivery was 27.1% and PNC was 18.4% [9]. Debre-markos, the capital city of East Gojjam zone, is located in a northwest direction, 299 km from the national capital Addis Ababa. According to a US Agency for International Development estimate, the population of East Gojjam zone was estimated to be 2,613,835, with a male population of 1,292,042 (49.4%) and a female population of 1,321,788 (50.6%) in 2017 [16].

### The context of the primary health care unit

The Ethiopian health service is structured into a three-tier system, with primary, secondary and tertiary levels of care. The primary level of care includes primary hospitals, health centers and health posts. The primary health care unit is composed of a health center and five satellite health posts. These provide both preventive and curative services to approximately 25,000 people altogether. Health centers serve as a referral center and practical training institution for health extension workers (HEWs). In turn, primary hospitals serve as a referral center for health centers under its catchment areas and a practical training center for nurses and other paramedic health professionals [17].

Ethiopia’s health-extension program is a community-based strategy to deliver health promotion, disease prevention and selected curative health services at the community level. It is a mechanism to provide health services free of charge to all segments of the population in the country by paid HEWs. HEWs are drawn from the community they are going to serve, and are trained for 1 year. Two HEWs per health post are deployed to provide services under the 16 packages by making house-to-house visits. Improving access and utilization of latrines, increasing contraceptive acceptance rates and ANC and PNC, improving health-seeking behavior, expanding vaccination services, improving malaria control and prevention, and reduction of new human immunodeficiency virus infection are some of the major packages that are expected to be delivered by HEWs. HEWs spend 75% of their time on home-to-home visits providing promotional and preventive interventions and 25% of their time at health posts [13, 17]. Even though maternal health services are delivered at different levels of the health system, this trial is focused on improving service delivery in the primary health care units. Using the existing referral linkages between primary health care units, all three contact points (ANC, delivery and PNC) are expected to happen at the health center level (as recommended) where the monitoring box is placed. If the mother made one of the visits, including delivery by her preference, at facilities other than the intervention health center it will be tracked by HEWs and, if it is confirmed, her individual folder will be transferred to the next stage. However, if the mother is permanently moved to another facility/residence, she will be excluded from the study. Since the services are similar for primary hospitals and above, the trial can also be applied to hospitals.

### Participant eligibility criteria

The participants for this trial will be pregnant mothers residing in three of the selected districts of East Gojjam zone (Debre-Markos, Gozamin and Machakel districts). Recruitment will pass a two-stage screening process. Using a preset criterion, the initial screening will be done at the community level using the pregnancy screening checklist from Stanback et al [18]. (last menstrual period for gestational age dating), and mothers having a suspected pregnancy will have a laboratory confirmation at the health center. Those having a gestational age of 16 weeks will be recruited for the study. Mothers who fulfill the above criteria but who have severe psychological illness which could interfere with consent and study participation, those who have severe clinical complications that need hospitalization and mothers who need a special type of ANC follow-up, other than the recommended focused ANC, will be excluded from the study.

### Sample size determination

The sample size was calculated based on the recommendations of sample size calculations for cluster randomized controlled trials with fixed number of clusters [19] by using the following assumptions: utilization of the third PNC in the control group is 16% based on a previous study [20], the number of clusters available is 30 (15 clusters per arm), with 95% confidence interval and 80% power, and an intracluster correlation coefficient of 0.04849 [21]. The sample size was calculated to determine the number of observations required per cluster for a two-sample comparison of proportions (using normal approximation) using STATA software. Assuming individual randomization, the sample size per arm is 194; allowing for cluster randomization, the average cluster size required is 40. A total of 1200 pregnant mothers (600 in the intervention and 600 in the control groups) will be recruited to detect a 12% increase in the utilization of ANC visit four, skilled birth attendance and PNC. The outcome is treated as a binary measure.

### Randomization and sampling procedures

Among the 16 districts in East Gojjam zone, Debre-Markos, Gozamin and Machakel were selected on purpose after confirming that each of them did not have any current projects aiming to increase the utilization of maternal health services. Health posts (with their catchment areas) under selected districts are the unit of randomization for this trial. Since a component of the planned intervention will be delivered at the community level, individual randomization is not appropriate in this situation as it would result in contamination. Thus, 30 nonadjacent health posts (clusters) were selected. Once cluster recruitment has been completed, participating clusters were allocated to one of the two trial arms (at 1:1 allocation) on an equal basis (a total of 30 clusters, fifteen clusters in each arm) using an SPSS-generated random number sequence. This trial aims to increase service utilization, and we used mothers who had previously delivered as a baseline to compare the status of service utilization in selected areas, as well as to compare the status of maternal health service utilization after the intervention. For this, all mothers who have given birth in the previous 1 year, regardless of their place of delivery, who are living in the catchment area of the selected health posts were entered in SPSS and ‘random case selection’ of the required sample size was performed.

### Screening and enrollment

In Ethiopia, the linkage between primary health care units is defined and each health post has its own catchment population to serve. This linkage works primarily by proximity of health facilities to the catchment population. Community-level referral of this trial works following this defined primary health care unit linkage. Based on this, HEWs will use a family folder (a detailed record about households) to identify women of reproductive age. The first-level screening will then take place at the community level using the pregnancy screening checklist of Stanback et al. [18]. Mothers having suspected pregnancy will receive a community-level referral slip for them to be received by the nearby health center. The second screening will take place at the health center using a beta-human chorionic gonadotropin (HCG) urine test as laboratory confirmation of pregnancy. Mothers who have known pregnancy but have not initiated the first ANC will also be referred for the service. If a mother has a positive HCG result, she will be recruited for the study and on the same day she will receive the first ANC service.

### Intervention description

#### Intervention

**Fig. 2:**
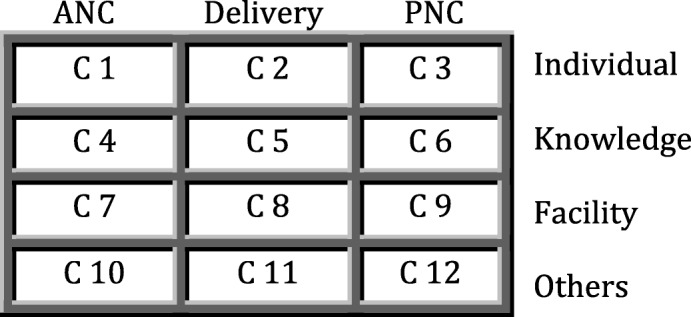
Representation of the 12-compartment health education scheduling box to be presented at the health post (C compartment). For example, if a mother misses antenatal care (ANC) following a knowledge gap, the reason-picking card will be placed in compartment 4. Compartments containing more cards will be a focus for future discussion. PNC, postnatal care

The intervention has two main focuses; first, creating demand at the community level performed by HEWs and, second, service utilization monitoring through dropout-tracing mechanisms. Two separately designed boxes were introduced to help facilitate the above interventions. Health posts received ‘health education scheduling boxes’. The main purpose of these 12-compartment boxes (Fig. 2) is to prioritize the most common reasons raised by mothers for not utilizing each of the maternal health services and to provide focused health education. As the Ethiopian health system encourages mothers to receive maternal health services at the health-center level and above, for these interventions HEWs have performed more demand-creation activities.

##### Health post

HEWs use family folders to locate women of reproductive age and carry out home-to-home pregnancy screening using the checklist of Stanback et al. [18]. Mothers having a suspected pregnancy will be referred to the nearby health center for laboratory confirmation and, if they are pregnant, they will receive the first ANC at the receiving health center. In this process, HEWs link mothers with the receiving health center through a referral slip.

While conducting home-to-home visits, HEWs identify reasons raised by women for not utilizing maternal health services (ANC, skilled birth attendance and PNC) and the list of reasons will be kept in health education scheduling boxes. Reasons raised by most mothers will be the topic for health education by HEWs. If a service is missed, HEWs will be informed and they will identify the reason with the mother and discuss it with her in attempt to get her to resume the service.

##### Health center

**Fig. 3:**
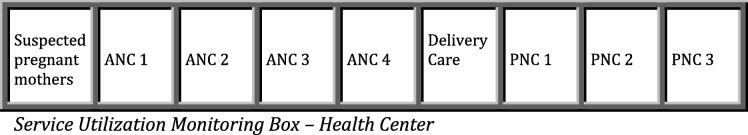
Representation of the service utilization monitoring box at the health center. The individual folder for the mother with a list of services completed for that specific visit will be placed in the correct compartment; when the mother attends for a subsequent service her folder will be transferred to the next compartment, but when a mother does not attend her folder will remain in the previous compartment. Using this process, the dropouts for each visit can be traced. ANC antenatal care, PNC postnatal care

Once the mothers are linked to a nearby health center, they will be followed for their subsequent attendance of consecutive maternal health care services of ANC (second to fourth visits), skilled birth attendance, and PNC (1–3 visits). This will be checked using a specially designed service utilization monitoring box. This box is a nine-compartment box (Fig. 3); the first compartment is for mothers suspected of being pregnancy using the pregnancy screening checklist from the HEWs. If the mothers arrive and confirm their pregnancy (via HCG test) they will receive the first ANC and their card will be transferred to the next compartment; the same procedure will be followed for the coming consecutive maternal health care service visits. The visits for ANC will follow the four-visit WHO ANC model (i.e., first ANC before 16 weeks of gestation, second ANC at 24–28 weeks, third ANC at 30–32 weeks, fourth ANC at 36–40 weeks); facility delivery is that assisted by skilled health-care providers at the health-center level and above; and visits for PNC are PNC visit 1 at 6 h, PNC visit 2 at 6 days and PNC visit 3 at 6 weeks after delivery. This service utilization monitoring box will help midwives identify mothers who fail to follow the above smooth transition. If a mother is identified as a ‘dropout’, midwives at health centers will communicate to HEWs in the area in which the mother lives. Again, these mothers will be approached for person-centered health education to resume their service within the recommended time interval.

#### Control

Participants in the control arm will receive existing routine care of mass health education by HEWs and will receive maternal health care services at health centers through being given appointments for subsequent follow-ups. Unlike the intervention arm, this group will not receive community-level pregnancy screening and will not be given access to a health education scheduling box, a person-centered health education manual or a service utilization monitoring box.

### Intervention process

Before the study team introduced the intervention, a detail intervention package was developed with a focus on narrating what steps to follow while implementing the intervention. In addition, other pre-introduction activities were undertaken, including development of a training manual and a person-centered manual for HEWs and health care providers (Amharic version), development of the specification for the health education scheduling boxes and service utilization monitoring boxes, and procurement of 15 boxes for the health posts and 5 boxes for the health centers. A sensitization workshop was conducted for local health administrators, along with training for all HEWs from 15 selected health posts and the training of 2 midwives from each selected health center (one delivery case team lead and one midwife). Boxes, printed checklist referral slips, registers and manuals were distributed accordingly, and onsite orientation was made. The community survey and enrollment was then started. Follow-up visits were made based on the previously developed compliance parameters.

### Data collection tools and techniques

Data will be collected using a semistructured and pretested questionnaire. The questionnaire used to collect the data was designed by reviewing relevant literature and related national and international standards, and the focus is on educational and ecological assessment, a mother’s experience related to pregnancy, delivery and PNC, social and epidemiological assessment, behavioral and environmental assessment, general social support and social capital and health service quality components. This will be translated to an Amharic version (the local language of the study area).

The translated version was uploaded to KoBo toolbox, and an open data kit (ODK) will be used to collect data. The study will use face-to-face interviews and the data will be collected using mobile devices in front of each selected mother’s home. Completed questionnaires will be saved in the mobile devices and will be sent to the central server immediately after completing the interview. In cases where the data collectors cannot complete the questionnaires and when an internet connection cannot be accessed the completed questionnaires will be forwarded when there is an internet connection available. The data collection will be managed directly from the Jimma University server. Each data collector and supervisor has a code to make data management easy. If a study participant in either arm moves from the study area before the study is completed, they will be registered as dropouts and lost to follow-up. This trial will have two phases of data collection: baseline study (before the intervention is introduced) and end-line study (after the intervention is introduced).

### Data quality control

Training for selected data collectors and supervisors will be conducted, with the focus on making every question commonly understood. In addition, a manual explaining each and every question with its response categories has been prepared; this manual also includes sections explaining ‘how to use ODK to collect data’ with examples demonstrating the features of the application. The data collection will be conducted by midwives who hold BSc. degrees, and will be supervised by two professionals who hold MPH degrees. In addition to this, a close follow-up by the principal investigator will be done. The field data collection will be supported by the ODK in which each and every question is required to be answered, helping to avoid missing data. Range checks will also be made for selected data values. The ODK will also allow editing of the responses in front of each interviewee. Similarly, global positioning system coordinates of each and every interview will be recorded at the end of each interview which will allow graphical representation of the study site.

### Analysis plan

Data from the trial will be analyzed by an intention-to-treat analysis at both cluster and participant levels. Participants will be assigned to the cluster they were resident in at the time the trial began. The primary data analysis will take place at the cluster level. Risk ratios will be computed at the cluster level, and the results of this cluster summary will be compared using *t* tests. Primary and secondary outcomes will be compared between intervention and control groups with random effects logistic regression models, taking account of clustering/correlation. All estimates will be presented with 95% confidence intervals. STATA version 13.0 will be used to run the analysis.

### Outcome measures

Primary outcomes are the continued utilization of maternal health care service (ANC to PNC), mothers attending the fourth ANC visit, mothers delivering at health facilities assisted by skilled care providers, and mothers attending the third PNC visit at 6 weeks after delivery.

Secondary outcome measures will assess whether the trial has an effect on early initiation of ANC (initiation of ANC before 16 weeks of gestation), and utilization of the first ANC (first ANC visit attendance, regardless of the time of initiation).

### Dissemination plan

A detailed study protocol will be published in an open-access journal. Any modifications made to the protocol will be communicated to relevant parties, such as the trial registry and both of the ethics committees. After the results are produced they will be submitted to the Jimma University, the Amhara Region Public Health Institute, the East Gojjam zone Health Department, and governmental and nongovernmental organizations working in maternal health programs. The findings of this study will also be presented in scientific conferences. Also, a manuscript will be prepared and submitted to a peer-reviewed scientific journal for possible publication.

## Discussion

This trial is designed to improve continued utilization of maternal health care services and will be applied both in urban and rural settings. Unlike routine care, pregnancy identification will take place at the community level. As this trial is designed to be implemented by combining both demand-creation and service utilization monitoring components, the investigators believe that the evidence generated will contribute to maternal health programs at the regional and national level (Additional files 1 and 2, Table 1).

## Trial status

Thirty clusters have been selected and the preparatory activities such as development of training manuals, referral slips and registers are completed. A sensitization workshop for local health administrators and training for intervention implementers (HEWs and midwives) has been conducted. The specification for specially designed boxes for health posts and health centers has been developed and procurement and distribution has been made accordingly. The baseline study has collected data in both the intervention and control clusters, and enrollment of mothers as per the criterion is being conducted. This is protocol version 1, dated 26 March 2019. Recruitment began on 22 November 2018, and the approximate date of recruitment completion is October 2019.

## Supplementary information

**Additional file 1.** SPIRIT 2013 checklist: recommended items to address in clinical trial protocol.

**Additional file 2.** Items for a WHO trial registration dataset (TRDS).

## Data Availability

Not applicable.

## Abbreviations

ANC: Antenatal Care
CBBSI: Checklist-based box system intervention
HCG: Human chorionic gonadotropin
HEW: Health extension worker
ODK: Open-data kit
PNC: Postnatal care
WHO: World Health Organization
