# Supplementary material for: Effectiveness of Checklist-Based Box System Interventions (CBBSI) versus routine care on improving utilization of maternal health services in Northwest Ethiopia: study protocol for a cluster randomized controlled trial
Source: Trials. 2020 Feb 7;21:151. doi: 10.1186/s13063-019-4002-3 (PMC7007673; doi:10.1186/s13063-019-4002-3)
Supplement: Supplementary file 1 — Additional file 1. SPIRIT 2013 checklist: recommended items to address in clinical trial protocol. [file 13063_2019_4002_MOESM1_ESM.doc]

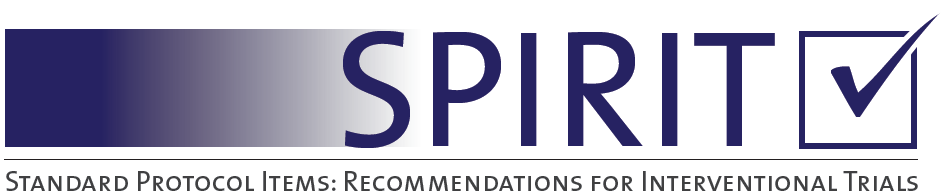


| Section/item | Item No | Description |
| --- | --- | --- |
| **Administrative information** | | |
| Title P-1 | 1 | Effectiveness of Checklist based Box system Interventions (CBBSI) versus routine care on improving utilization of Maternal Health Service in North West, Ethiopia: Cluster Randomized Controlled Trial |
| Trial registration  P-2 | 2a | ClinicalTrials.gov NCT03891030, 26 March 2019, Retrospectively Registered |
|  | 2b | Items from the World Health Organization Trial Registration Data Set included in the protocol |
| Protocol version P-13 | 3 | Version 1 dated 26 March 2019 |
| Funding  P-14 | 4 | The study has got financially support from Armauer Hansen Research Institute (AHRI) |
| Roles and responsibilities  P-15 | 5a | **Netsanet Belete**, Jimma University - initiated the trial idea, wrote initial draft of the proposal, developed the protocol and supervises the implementation of the intervention, study & statistical design  **Mulusew Gerbaba**, Jimma University - participating in maturing the implementation plan, study design and statistical design of the trial  **Gurmesa Tura**, Jimma University - participating in maturing the implementation plan, study design and statistical design of the trial |
|  | 5b | Jimma University, Jimma Ethiopia, PO Box-384 |
|  | 5c | AHRI had no role in the design of this study and will not have any role in the data collection, management, analysis, and interpretation of data; writing of the report; and the decision to submit the report for publication |
|  | 5d | PhD and MSc holder reproductive health professionals centrally oversee the study; data collection is managed directly from the central server of jimma university. |
| Introduction  Background and rationale P-2 | 6a | Maternal mortality is still high in Ethiopia. Antenatal care, use of skilled delivery and postnatal care are key maternal health care services that can significantly reduce maternal mortality. |
|  |  | However, in low and middle income countries including Ethiopia, utilization of these key maternal health care services (MHCS) are not enough to deliver the recommended preventive, promotive and curative services during pregnancy, delivery and the post natal period.  Most of the studies conducted in continued utilization of maternal health care (from antenatal care to post natal care) showed that: service utilization decreases as mothers goes from the pregnancy care to post-pregnancy care. A study conducted in Nepal showed that 47.7% attended at least one and 30.7% attend at least four ANC visits. A study assessing PNC utilization in Pakistan also showed that, around 70% did not consult with anyone for PNC and majority (70%) mentioned that postnatal check-up was not necessary. Comparison of findings from studies investigating utilization of MHCS in low and middle-income countries indicates that women are more likely to attend ANC than to deliver in health facilities. For instance, in Ethiopia, overall MHCS utilization is low, 2016 EDHS showed that 62% and 32% of pregnant women made at least one and four ANC visits; SBA and PNC are reported as 27.7% and 16.5% respectively. Regarding the timely initiation of antenatal care, 2014 Ethiopian Mini DHS showed that the average time the women start the first ANC was five month. So, it can be shown that, even though effective health interventions are available, most studies in developing countries like Ethiopia indicate that the potential to deliver health information and services using MHCS still remains underutilized.  In general, timely initiation and continued utilization of MHCS is implemented far from the international recommendation, and the proportion of mothers utilizing the service decreases as one go from the first ANC to the third PNC. Thus, the aim of this trial is to use the rigor of Cluster Randomized Controlled Trial for the evaluation of checklist based box system interventions on improving MHCS utilizations, which will basically intervene on the timely initiation and continued utilization of maternal health care services. Antenatal care service utilization will follow the world health organization four-visit ANC model/ focused antenatal care. |
|  | 6b | Routine care service for maternal health service delivery is being implemented for over years, but even the regularly conducted demographic and health survey in the trial country, Ethiopia showed a slight improvement over years. |
| Objectives  P-4 | 7 | **Primary Objective** – To assess the effectiveness of CBBSI on continued utilization of maternal health care service, (antenatal care 1^st^ -4^th^, institutional delivery assisted by skilled health care providers, and postnatal care 1^st^-3^rd^).  **Secondary Objectives –** secondary outcome measures will assess whether the trial has an effect on the following maternal health service utilization;   - Early initiation of antenatal care (before 16 weeks of gestation) - Attending fourth antenatal care - Utilization of facility delivery - Attending the third postnatal care |
| Trial design  P-4 | 8 | Checklist based box system intervention trial is designed as a Randomized, Controlled, double blind, parallel group with 1:1 allocation. |
| Methods: Participants, interventions, and outcomes | | |
| Study setting  P-4 | 9 | The trial is implemented in East gojjam zone, Northwest, Ethiopia. East gojjam zone, zonal health department was communicated to obtain list of districts with in the zone. Of those, Debre-Markos, Gozamin and Machakel Districts were selected. 30 health posts with their catchment population (cluster units) and 10 health centers were selected for the trial; these are recruited from urban and rural settings. |
| Eligibility criteria  P-5 | 10 | The participants for this trial will be pregnant mothers under 16 weeks of gestation. A two-stage enrolment process: initial screening using Stanback et al, 1999 and a laboratory confirmation of pregnancy at health center will be employed. Mothers having 16 weeks of gestational or less will be recruited. Mothers who fulfil the above criteria but, who have severe psychological illness, which could interfere with, consent and study participation, those who have sever clinical complications that need hospitalization and mothers who need special type of ANC follow-up, other than focused ANC will be excluded from the study. |
| Interventions  P-7 | 11a | **Trial Arm I (Intervention)**  The intervention has both community level (behavioural change) and facility level (service utilization drop out tracing mechanism) components. The list of Women of reproductive age will be obtained from the family folder, and based on that home to home visit will be made by HEWs. While making these visits, they will do pregnancy screening using Stanback et al, 1999 checklist. Mothers having a suspected pregnancy will receive a referral slip to be received by the nearby health center and a copy will remain with health extension workers. In addition, these home-to-home visits will allow health extension workers to identify individual reasons for not utilizing the service. List of mothers referred will be registered with a special referral registry with the expected date of health center visit and the revisit day (for mothers not visiting HCs). The community level behavioural change component will focus on; approaching pregnant and delivering mothers for person-centered problem solving health educations. Beyond this individual based health education, the work of HEWs, identifying underlying reasons for not utilizing the service will help HEWs to make decisions by using locally generated data. This is done through a special designed health education scheduling boxes placed at health posts. Of the reasons identified, for not utilizing MHCS, reasons raised by most of the mothers will be used as a topic of discussion during platforms like, pregnant mothers conference.  Once the mothers are linked to the nearby health center, they will be followed for their subsequent attendance of consecutive maternal health care services (ANC (2-4^th^ visits), skilled birth attendance, and post-natal care (1-3)). This will be checked using special designed service utilization monitoring box (procured and given to intervention health centers). This box is a nine-compartment box: the first compartment is for mothers suspected for having pregnancy using the pregnancy-screening checklist. If the mothers came and confirm their pregnancy (HCG test) they will get the first ANC and their card will be transferred to the next compartment, the same procedure will be followed for the coming consecutive MHCS visits. The visits for ANC service will follow the 4-visit WHO ANC model (i.e. First ANC (before 16 weeks of gestation), second ANC (24-28 weeks), Third ANC (30-32 weeks), fourth ANC (36-40 weeks), and facility delivery assisted by skilled health care providers (health canter and above) and PNC 1 at 6 hours, PNC 2 at 6 days and PNC 3 at 6 weeks of delivery). This service utilization-monitoring box will help Midwives to identify mothers who fail to follow the above smooth transition. If a mother is identified as a ‘dropout’, Midwives at health centers will communicate health extension worker in which the mother is belonging. Again these mothers will be approached for person-centered health education to resume their service within the recommended time interval. |
|  | 11b | **Trial arm II (control)**  Participant in the control arm will receive the existing routine care: mass health education by health extension workers and receive MHCS at health centers through giving appointments for subsequent follow-ups. Unlike the intervention arm, this group will not receive community level pregnancy screening survey, will not given health education scheduling box, person centered health education manual for health posts and service utilization monitoring box for health centers.  For those raising distance as a barrier (those living far from the recruiting health center), except the first ANC, delivery care and the first PNC), the remaining health services can be given at health posts. This will be done with effective communication with health center midwives. |
|  | 11c | Regular review of service utilization by midwives working in health centers will be done, so that mothers dropping from utilizing the recommended service can be identified easily. |
|  | 11d | Maternal health service increment interventions, other than checklist based box system interventions, are excluded during the recruitment of clusters. |
| Outcomes  P-12 | 12 | **Primary Outcomes –** Proportion of mother attending continued utilization of maternal health services (Antenatal care 1^st^ -4^th^, facility delivery and postnatal care 1^st^ -3^rd^) in the intervention and control arms  **Secondary Outcome –** Proportion of mothers attending the first ANC care before 16 weeks of gestation in the intervention and control arms  Proportion of mothers attending the fourth ANC care in the intervention and control arms  Proportion of mothers attending facility delivery in the intervention and control arms  Proportion of mothers attending the third post-natal care in the intervention and control arms |
| Participant timeline  P-9 | 13 | A schematic diagram representing enrolment, intervention and assessment is annexed with this document & included in the main text of the study protocol (Annex 1). |
| Sample size  P-6 | 14 | The sample size was calculated using the recommendations for sample size calculations for cluster randomized controlled trials with fixed number of clusters. The following assumptions were considered: to detect an increase of postnatal care three utilization from 16% to 28%, number of clusters available-30, with 95% confidence interval and 80% power, intra-cluster correlation coefficient of 0.04849, 15 clusters per arm. The sample size was calculated to determine number of observations required per cluster, for two-sample comparison of proportions (using normal approximation), Then allowing for cluster |
| Recruitment P-7 | 15 | randomization, average cluster size required is 40, and the final sample size is 1200 pregnant mothers (600 in intervention, and 600 in control).  Community level survey is the main strategy to recruit pregnant mothers. Conducting community level survey regularly will be used as an additional strategy to achieve adequate sample size for the study. |
| **Methods: Assignment of interventions (for controlled trials)** | | |
| Allocation: |  |  |
| Sequence generation P-7 | 16a | Clusters were randomly assigned to either intervention to control group in 1:1 allocation, based on random case selection command of SPSS. |
| Allocation concealment mechanism P-6 | 16b | This trial employees cluster randomization. Unlike individual randomization, the allocation is done once and as a concealment strategy we used random selection command of SPSS. |
| Implementation  P-10 | 16c | After cluster recruitment was completed, the central team did allocation. Participant enrollment is being done in two steps: Initial screening is being done by health extension workers (community level health workers), then midwives at health centers using laboratory tests will confirm the diagnosis. Those recruited from intervention clusters will receive the intervention and those recruited from the control arm will receive the routine care. |
| Blinding (masking)  P-4 | 17a | Trial participants and outcome assessors will be blinded. This trial uses professionals who were not participated in delivering the intervention as an outcome assessor. Blinded outcome assessors will also send collected data directly to the central. |
|  | 17b | The study team is planned to maintain the blinding as far as possible, but if un-blinding is deemed necessary this will be reported. |
| **Methods: Data collection, management, and analysis**  Data will be collected using a semi-structured and pre-tested questionnaire, focusing on educational and ecological assessment, pregnancy, delivery and postnatal care related, social and epidemiological, behavioural and environmental assessment, general social support and social capital and health service quality components. This was translated to Amharic version and uploaded to kobo toolbox to collect data using ODK. Filled questionnaires will be sent to the central server. Training for data collectors and supervisors will be conducted, with the focus of making common understanding for each and every question. | | |
| Data collection methods P-10 | 18a | In addition, manual explaining each and every question with its response categories, including ‘how to use ODK to collect data’ with examples demonstrating the features of the application was prepared. B.Sc. holder midwifes will do the data collection and supervised by MPH holder professionals. |
|  | 18b | Once pregnant mothers are recruited, reasonable efforts will be made to follow the mother throughout the study period, since keeping mothers under the umbrella of health care, until the third PNC is also the main objective of the intervention. |
| Data management  P 10 & 11 | 19 | The data collection will be managed directly from Jimma university server. Each data collector and supervisor has got a code; this will make data management easily done. The field data collection will be supported by ODK in which each and every question is settled to be required; help to avoid missing data. Also range checks were setted for selected data values accordingly. This ODK will also allow editing the responses in front of each interviewee. Similarly GPS coordinates of each and every interview place will be recorded at the end of each interview, this will allow to graphically represent the study site. |
| Statistical methods  P-12 | 20a | Data from the trial will be analysed by intention to treat analysis at both cluster and participant levels. Participants will be assigned to the cluster they were resident in at the time the trial began. Data analysis will take place in two levels, in cluster level and in individual level. Risk ration will be computed at cluster level, and the results of this cluster summary will be compared using t-test. Primary and secondary outcomes will be compared between intervention and control groups with random effects logistic regression models, taking account of clustering/correlation. All estimates will be presented with 95% confidence intervals. |
|  | 20b | Generalized Estimating Equation (GEE). |
|  | 20c | Per-protocol analysis will be conducted. |
| **Methods: Monitoring** | | |
| Data monitoring  P-11 | 21a | The study team will do data collection monitoring centrally. |
|  | 21b | The central study team will do all the baseline and end line analysis and decisions to terminate the trial. |
| Harms P-14 | 22 | Not Applicable |
| Auditing P-10 | 23 | A compliance parameter was developed for both the behavioural and facility component and the intervention is being followed using that. |
| Ethics and dissemination | | |
| Research ethics approval P-14 | 24 | The institutional review board of jimma university and Amhara Public Health Institutions reviewed and approved the protocol and informed consent template. |
| Protocol amendments | 25 | Any modifications that will be made to the protocol will be communicated to relevant parties such as trial registry and both of the ethics committees. |
| Consent or assent | 26a | Data collectors will obtain oral informed consent. |
|  | 26b | Not Applicable. |
| Confidentiality | 27 | All study-related information will be stored securely at the study site. All participant information will be stored in a limited access places at the study site. All study databases will be secured with password-protected access systems, which will be accessed from the sponsor organization. |
| Declaration of interests P-14 | 28 | The authors declare that they have no competing interests. |
| Access to data | 29 | The central study team has the permission to access the dataset. |
| Ancillary and post-trial care | 30 | Not Applicable. |
| Dissemination policy P-14 | 31a | Trial protocol was registered at ClinicalTrials.gov. Detailed study protocol will be published on open access journal. After the result is produced, it will be submitted to Jimma University, Amhara public health institute, East Gojjam zone health department and governmental and non-governmental organizations working in maternal health programs. The findings of this study will also be presented in scientific conferences. Also, manuscript will be prepared and submitted to a peer-reviewed scientific journal for possible publication. |
|  | 31b | Both the protocol and findings will be drafted and approved by the study team. |
|  | 31c | We will deliver completely de-identified dataset not later than 12 months of end line study. |
| Appendices |  |  |
| Informed consent materials | 32 | Participant information sheet and consent form are annexed (annex 2). |
| Biological specimens | 33 | Not Applicable. |

**Annex 1: Participant Flow**

**Randomization**

**Allocation**

71 Health Posts three districts

30 Health posts selected

Informed Consent and Baseline Data Collection

Informed Consent and Baseline Data Collection

15 Health Posts/Intervention

15 Health Posts/Control

Enrolment

Eligibility Screening

Eligibility Screening

Intervention group Receives CBBSI

Control group Receives Routine care

Intervention

10 Months Follow-up Survey

10 Months Follow-up Survey

Informed Consent and End-line Survey

Informed Consent and End-line Survey

Analysis

Analysis

*Fig1. Participant Flow*

**Annex 2: Participant Information and Consent form**

**Participant Information Sheet**

**Research Project Title**

Effectiveness of checklist based box system interventions on improving maternal health care service utilization:

**Introduction**

Good morning/afternoon! My name is _________________. I am the member of the research team from Jimma University. We are speaking with mothers about their experience of during pregnancy, delivery and the post-partum period.

**Purpose of the Research**

This research aims to investigate the effectiveness of checklist based box system on improving utilization of maternal health care services: antenatal care, health facility delivery and post-natal care. This research will investigate the effects of the aforementioned intervention of person-centered health education at community and health post level and service utilization monitoring box implementation at health center on the improvement of service utilization.

**Research procedure**

We have selected mothers to participate in this study by chance. You are among those selected mothers to be part of this study. I will ask you questions concerning personal, socio-demographic and your experience during your last pregnancy, delivery and the post-partum period, social and epidemiological experiences, behavioural and environmental related, general social support and social capital and health service quality questions. I will also ask the information you have regarding the knowledge and health service seeking behaviour you had during your last pregnancy. The interview will take about 40 minutes. So, I kindly request your volunteer participation for this study.

**Possible Disadvantage and Risks**

Participating in this research is not anticipated to cause you any disadvantages or discomfort. If you feel discomfort sharing personal information, you can refuse answering.

**Benefit**

Whilst there are no immediate benefits for those people participating in the project, it is hoped that this work will have a beneficial impact on knowing the effectiveness of the aforementioned intervention on its contribution of improving utilization of maternal health care services.

**Confidentiality**

All the information that we collect about you during the course of the research will be kept strictly confidential. You will not be able to be identified or identifiable in any reports or publications. Any data collected about you in the data collection device will be stored online in a form protected by passwords. Data collected may be shared in an anonymised form by the research team. These anonymised data will not allow any individuals or their institutions to be identified or identifiable.

**Refusal/Withdrawal**

Your right as a study participant is respected; you can refuse to answer any question to which you are not comfortable. You may stop participating in the interview at any time if not convenient for you without losing any of your rights as a participant. However, your active participation and genuine responses have paramount importance in improving maternal health care services in future.

**Informed Consent Agreement Form**

**English Version**

Good Morning/Good Afternoon: My name is (name of the data collector). I kindly invited you to participate in the study investigating the effectiveness of checklist based box system on maternal health service utilization improvement. This study is conducted by Jimma University and financially supported by Armauer Hansen Research Institute (AHRI). The finding of the study will be used as an input to for the project assessing the effectiveness of checklist based box system implementation on the improvement of maternal health care service utilization. You will not get any risk by participating on this study, but your response together with the response of others will be used as an input for the project aiming to improve the utilization of maternal health care services. If you don’t want to continue with responding questions, you may refuse to answer questions at any time. If you need further information about the study, you can contact a representative of the research team, Netsanet Belete [Tel. - +251-939-935135, E-mail – netsanetb2009@gmail.com]

With due understanding of the aforementioned information, are you willing to participate in the study?

Yes 🡺Continue

No 🡺End
