# Supplementary material for: Effectiveness of Checklist-Based Box System Interventions (CBBSI) versus routine care on improving utilization of maternal health services in Northwest Ethiopia: study protocol for a cluster randomized controlled trial
Source: Trials. 2020 Feb 7;21:151. doi: 10.1186/s13063-019-4002-3 (PMC7007673; doi:10.1186/s13063-019-4002-3)
Supplement: Supplementary file 2 — Additional file 2. Items for a WHO trial registration dataset (TRDS). [file 13063_2019_4002_MOESM2_ESM.docx]

| **Items from WHO Trial Registration Data set (TRDS)** | |
| --- | --- |
|  |  |
| **Data Category** | **Information** |
| Primary registry and trial identifying number | ClinicalTrials.gov NCT03891030 |
| Date of registration in primary registry | 26 March 2019 |
| Secondary identifying numbers | CBBSI/2019 |
| Source(s) of monetary or material support | Armauer Hansen Research Institute (AHRI) |
| Primary sponsor | Jimma University |
| Secondary sponsor(s) | Armauer Hansen Research Institute (AHRI) |
| Contact for public queries | Aklog Nigussie (BSC), aklog.nigussie@gmail.com |
| Contact for scientific queries | Netsanet Belete (MSc), Jimma University, Jimma Ethiopia |
| Public title | Checklist Based Box system Interventions on improving maternal health care service |
| Scientific title | Effectiveness of Checklist based Box system Interventions (CBBSI) versus routine care on improving utilization of Maternal Health Service in North West, Ethiopia: Cluster Randomized Controlled Trial |
| Countries of recruitment | Ethiopia |
| Health condition(s) or problem(s) studied | Maternal Health Service Utilization |
| Intervention(s) | Intervention - Checklist Based Box system interventions |
|  | Control - Routine maternal health care |
| Key inclusion and exclusion criteria | Age - 15-49 Years, Females are eligible for the study |
|  | Inclusion Criteria - Confirmed pregnant mothers less than 16 weeks of gestation |
|  | Inclusion Criteria - severe psychological illness, sever clinical complications and hospitalized, mothers who are out of focused antenatal care |
| Study type | Interventional |
|  | Allocation: Randomized, Interventional model: parallel assignment, double masking |
|  | Primary Purpose: Behavioral change and service utilization |
|  | Phase II |
| Date of first enrolment | October 2018 |
| Target sample size | 1,200 |
| Recruitment status | Recruiting |
| Primary outcome(s) | Continued maternal Health Care service Utilization (ANC 1st - PNC 3) |
| Key secondary outcomes | Early Initiation of ANC (before 16 weeks of gestation) |
|  | Attending the fourth ANC |
|  | Attending Facility delivery |
|  | Attending the third PNC |
